# Clinical and Economic Case for Patient-Specific Total Knee Arthroplasty: A Prospective Study

**DOI:** 10.7759/cureus.80270

**Published:** 2025-03-08

**Authors:** Eli Johnson, Ethan Cottrill, Tara Mann, James Willey, Cambre Kelly, Daniel Dunaway

**Affiliations:** 1 Department of Neurosurgery, Duke University School of Medicine, Durham, USA; 2 Department of Orthopedics, Duke University Medical Center, Durham, USA; 3 Department of Clinical Affairs, restor3d, Inc., Durham, USA; 4 Department of Orthopedic Surgery, Far Oaks Orthopedists, Kettering, USA

**Keywords:** cruciate-retaining tka, economic impact, patient-specific, total knee arthroplasty, total knee replacement (tkr)

## Abstract

Introduction: Total knee arthroplasty (TKA) is a common surgical intervention for severe knee arthritis resulting in joint or cartilage destruction, with success largely depending on prosthetic design and surgical technique. This study compares two Cruciate Retaining (CR) TKA systems, the iTotal® Identity CR system (ConforMIS; Wilmington, MA), a patient-specific knee (PSK) system, with the Triathlon® Total Knee System (Stryker; Kalamazoo, MI), a standard off-the-shelf (OTS) system, hypothesizing that the PSK system results in better clinical outcomes and reduced costs.

Methods: This single-center, prospective study included 188 patients (217 knees) who underwent TKA with either the PSK or OTS system from August 2017 to February 2021. Participants were enrolled if they had a clinical indication for total knee replacement and were deemed suitable candidates for either system. All participants were adults (18+ years) and capable of providing informed consent. Data on operative times, complications, patient-reported outcomes, and cost metrics were collected and analyzed.

Results: Compared to the OTS group, the PSK group had a significantly shorter average total operating room (OR) time (85.2 vs. 95.9 minutes, p < 0.001), tourniquet time (50.2 vs. 64.3 minutes, p < 0.001), OR instrument setup time (5.0 vs. 11.9 minutes, p < 0.001) and OR instrument tear down time (1.5 vs. 7.0 minutes, p < 0.001). At preoperative baseline, the PSK participants had a worse mean Knee injury and Osteoarthritis Outcome Score for Joint Replacement (KOOS-JR) score (40.73 vs. 49.6, p <0.001) and worse Knee Society Scores (KSS) (50.9 vs. 62.7, p <0.001) compared to the OTS group. Finally, the PSK group demonstrated greater mean improvement postoperatively in both the KOOS-JR (37.33 vs. 24.61, p <0.001) and the KSS (113.1 vs. 98.1, p <0.001) scores, compared to the OTS group.

Conclusions: The PSK system demonstrated advantages in operative efficiency and improved patient-reported outcomes at the three-month follow-up compared to a standard OTS knee replacement system. These findings highlight potential benefits in adopting patient-specific implants in TKA, with implications for both clinical outcomes and healthcare cost savings. However, further research with longer follow-up periods in diverse patient populations is necessary to fully understand the long-term implications of patient-specific knee replacement systems.

## Introduction

Total knee arthroplasty (TKA) is a widely performed surgical procedure that aims to alleviate pain and restore function in patients with severe knee arthritis or injury, by reconstructing the knee joint [1]. TKAs have been shown to be effective, long-lasting procedures with good implant survivorship, with 82% of TKAs lasting at least 25 years [1]. However, despite the excellent survivorship of these implants, there is still a large percentage of patients who remain dissatisfied with their results [2].

TKA procedures are projected to increase significantly for the foreseeable future, from approximately 481,000 procedures per year in 2019 to more than 1,200,000 annually by 2040 and 2,900,000 annually by 2060 [3]. As the incidence of TKAs increases, there is a growing concern surrounding the economic burden of knee replacements on the healthcare system [4]. Medicare, for example, the primary payor for >60% of total joint arthroplasty procedures, is expected to face approximately $50B in annual expenditures for total joint arthroplasties by 2030 [5]. Curiously, there is significant variation in the cost of TKAs, driven primarily by differences in personnel and supply costs [4]. To address this latter variable, companies have recently begun to introduce new delivery and management models to reduce the total cost of TKA, such as through changes in the method of delivery, utilization of single-use instruments, and more efficient instrumentation [6,7].

The patient-specific knee (PSK) system utilizes a “just-in-time” delivery model coupled with single-use patient-specific instruments and implants. Thus, this system has the potential to reduce the economic burden to hospitals at multiple levels of the TKA procedure when compared to an off-the-shelf (OTS) TKA system that utilizes an inventory-based delivery model, coupled with a limited number of sizing options available to the surgeon during surgery. Given the projected increase in healthcare utilization and associated costs, the primary objective of this study is to compare the procedure-related costs of the PSK system to an OTS system for TKA and compare differences in clinical benefits. This evaluation will allow healthcare systems to make informed decisions when selecting products that can improve efficiency through reduced time and cost.

## Materials and methods

Study design and setting

This research was structured as a prospective, single-center study in the United States (Dayton, Ohio, USA) with the objective of comparing two cruciate-retaining (CR) total knee arthroplasty systems: a PSK system and a standard OTS system. Surgeons implanting both total knee arthroplasty systems utilized manual TKA rather than robot-assisted TKA techniques. The study took place over a period from August 2017 to February 2021.

Participants

Patients scheduled to undergo CR-TKA were enrolled consecutively by surgeons implanting either a PSK system or OTS system. After meticulous medical chart review and prior to obtaining informed consent, study participants were carefully selected based on specific inclusion and exclusion criteria to ensure the integrity of the research. Individuals were included if they had a clinical indication for total knee replacement and were deemed suitable candidates for either the PSK system or OTS system. Indications for TKA included osteoarthritis, rheumatoid arthritis, trauma, avascular necrosis, knee instability and deformity. Additionally, all participants were required to be adults over the age of 18 years and capable of providing informed consent. The process of consent was conducted in accordance with the guidelines set forth by the Institutional Review Board and good clinical practice, ensuring that participants were fully informed about the study's purpose, procedures, potential risks, and benefits before voluntarily agreeing to participate.

Conversely, the study excluded any individuals who were concurrently participating in another clinical study to prevent potential confounding of results and maintain the scientific validity of the study's outcome data. The exclusion of such individuals was a necessary step to ensure that the results reflected the true effects of the knee replacement options being studied, without interference from other clinical interventions or research protocols.

Variables

Patient characteristics included age, body mass index (BMI), gender, medical comorbidities, and medication use. Operative data included laterality, number of operating tool trays used, total operating room time defined as wheels in to wheels out, duration of time from tourniquet application to removal, instrument setup time on the sterile table before surgery, and sterile instrument tear down time on the sterile table.

Primary outcomes included procedure-related and device-related complications, operative times, and patient-reported outcome measures. The patient reported outcomes were Knee injury and Osteoarthritis Outcome Score for Joint Replacement (KOOS-JR) and the Knee Society Score (KSS). Secondary outcomes included post-discharge healthcare utilization, including readmission rates and the number of additional hospital/physician visits related to knee replacement. The study was comprised of preoperative baseline data, intraoperative and hospital stay data, as well as post-operative follow-up at 10-14 days to assess wound healing and signs of infection and 90 days to evaluate patient reported outcomes. Data were sourced from patient medical records.

Bias

To reduce selection bias and to accurately characterize the patient population, age at surgery, American Society of Anesthesiologists (ASA) score, gender, laterality of surgery, preoperative deformity, comorbid conditions, and Body Mass Index (BMI) were used to assess how well matched the cohorts were.

Study size

The sample size was selected based on the availability of participants during the enrollment period rather than a predetermined power analysis. A post-hoc power analysis revealed that a per group sample size of 30 to 38 participants would be needed to achieve a clinically significant effect size of 10 for patient reported outcome measures (KOOS-JR and KSS), at an alpha of 0.05 and power of 0.80, using Cohen’s d for independent sample t-tests.

Quantitative variables

Continuous variables were analyzed using MiniTab® Version 17 (State College, PA), with no imputations made for missing observations. These variables were summarized with standard descriptive statistics, including sample size, mean, standard deviation, median, minimum, maximum, and 95% confidence intervals. Categorical variables were summarized with frequency counts, percentages, and 95% confidence intervals.

Statistical methods

Demographic variables were summarized using descriptive statistics. Continuous variables were compared between treatment groups using independent t-tests (or Welch’s t-test for unequal variances), while categorical variables were analyzed using either Fisher’s exact test or a Chi-square test.

Devices

The study compared two types of CR-TKA devices: the patient-specific iTotal® CR system (ConforMIS; Wilmington, MA) and the OTS Triathlon® Total Knee System (Stryker; Kalamazoo, MI).

Ethical considerations

This study was approved by the Western Institutional Review Board (Study No. 1175797, WIRB Protocol No. 20171188). Participants were enrolled only after meeting the study criteria and providing informed consent, ensuring ethical compliance and respect for patient autonomy throughout the research process. Patients who elected not to participate in the research study were given the option to proceed with TKA, minimizing coercion.

## Results

Patient characteristics and operative data

**Figure 1:**
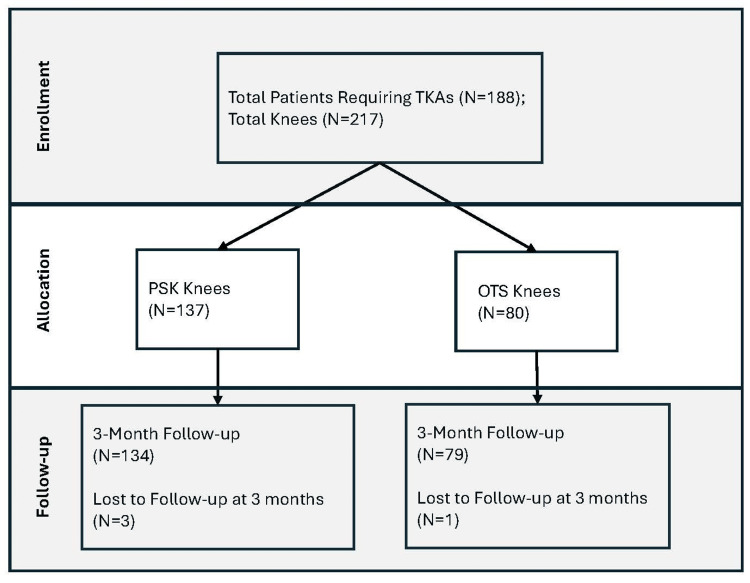
Study characteristics. OTS, Off-the-shelf; PSK, Patient-specific Knee; TKA, Total knee arthroplasty.

In our single-center prospective study, a total of 188 patients (217 knees) underwent TKA with either the PSK or the OTS System (Figure 1).

Among these, 137 knees received the PSK device, and 80 knees received the OTS device. The average patient age was 75.3 years, with a lower average age in the PSK group compared to the OTS group (73.8 vs. 77.8 years, p < 0.001). The average Body Mass Index (BMI) was comparable between the two groups, with the PSK group having an average BMI of 33.0 kg/m2 and the OTS group having an average BMI of 32.0 kg/m2 (p = 0.297). Preoperative Tylenol use was lower among the PSK group compared to OTS group (28% vs. 38%, p < 0.001). Aside from Tylenol, all other preoperative medication use, comorbidities and average ASA scores were not significantly different between the groups. These data are summarized in Table 1.

**Table 1:** Patient characteristics. This table is based on patient-level data, four patients did not provide comorbidity, medication, and ASA physical score data. ASA, American Society of Anaesthesiologists; BMI, Body Mass Index; COPD, Chronic obstructive pulmonary disease; df, degrees of freedom; NSAIDs, Non-steroidal anti-inflammatory drugs; OTS, Off-the-shelf; PSK, Patient-specific knee; SD, Standard Deviation.

	PSK (N=120)	OTS (N=68)	Total (N=188)	X^2^(df) = value, P-value	Effect size
Demographics					
Average age in years (SD)	73.8 (8.53)	77.8 (6.45)	75.3 (8.06)	<0.001	
Average BMI (SD)	33.0 (6.15)	32.0 (5.96)	32.6 (6.08)	0.297	
Gender				X^2^(1)=0.83, p=0.362	0.066
-Male n (%)	35 (29.2)	25 (36.8)	60 (31.9)	-	-
-Female n (%)	85 (70.8)	43 (63.2)	128 (68.1)	-	-
Comorbidities (n)					
Hypertension (%)	67 (55.8)	35 (51.5)	102 (54.3)	X^2^(1)=0.18, p=0.671	0.031
Hypercholesteremia (%)	49 (40.8)	18 (26.5)	67 (35.6)	X^2^(1)=3.30, p=0.0692	0.133
Diabetes (%)	18 (15.0)	16 (23.5)	34 (18.1)	X^2^(1)=1.59, p=0.207	0.092
Depression (%)	18 (15.0)	4 (5.88)	22 (11.7)	X^2^(1)=2.67, p=0.103	0.119
Malignancy (%)	6 (5.00)	8 (11.8)	14 (7.45)	X^2^(1)=1.93, p=0.159	0.108
Ischemic heart disease (%)	10 (8.33)	4 (5.88)	14 (7.45)	X^2^(1)=0.11, p=0.744	0.025
COPD (%)	10 (8.33)	4 (5.88)	14 (7.45)	X^2^(1)=0.11, p=0.744	0.024
Medication use (n)					
NSAIDS (%)	70 (58.3)	44 (64.7)	114 (62.5)	X^2^(1)=0.5, p=0.481	0.051
Tylenol (%)	28 (23.3)	38 (55.8)	66 (39.8)	X^2^(1)=18.78, p<0.001	0.316
Aspirin (%)	29 (24.2)	12 (17.7)	41 (19.5)	X^2^(1)=0.73, p=0.392	0.062
Lyrica (%)	15 (12.5)	4 (5.88)	19 (14.1)	X^2^(1)=1.43, p=0.232	0.087
Tramadol (%)	13 (10.8)	4 (5.88)	17 (10.9)	X^2^(1)=0.76, p=0.383	0.064
Hydrocodone (%)	8 (6.67)	4 (5.88)	12 (8.59)	X^2^(1)=0.0, p=0.998	0.000
ASA physical score					
Average ASA score (SD)	2.62 (0.58)	2.66 (0.54)	2.63 (0.56)	0.592	
ASA score II (%, df=2)	52 (43.3)	25 (36.8)	77 (41.0)	-	-
ASA score III (%, df=2)	62 (51.7)	41 (60.3)	103 (54.8)	-	-
ASA score IV (%, df=2)	6 (5.00)	2 (2.94)	8 (4.26)	-	-

**Figure 2:**
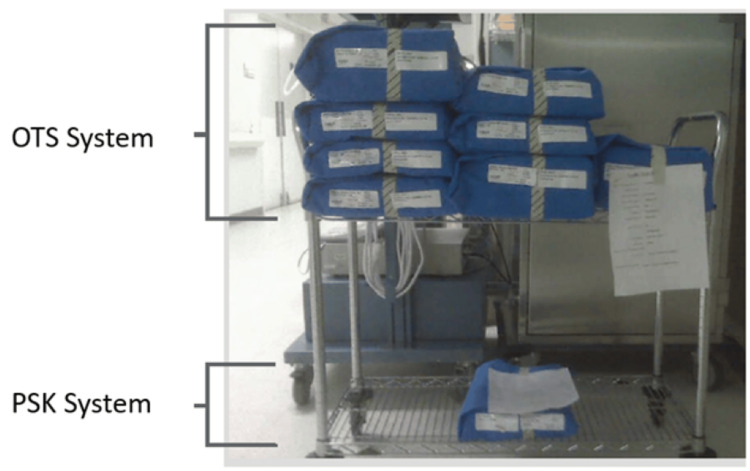
Operating room tray comparison. The OTS system requires six to eight compared trays to a single tray used by the PSK system. OTS, Off-the-shelf; PSK, Patient-specific knee.

**Figure 3:**
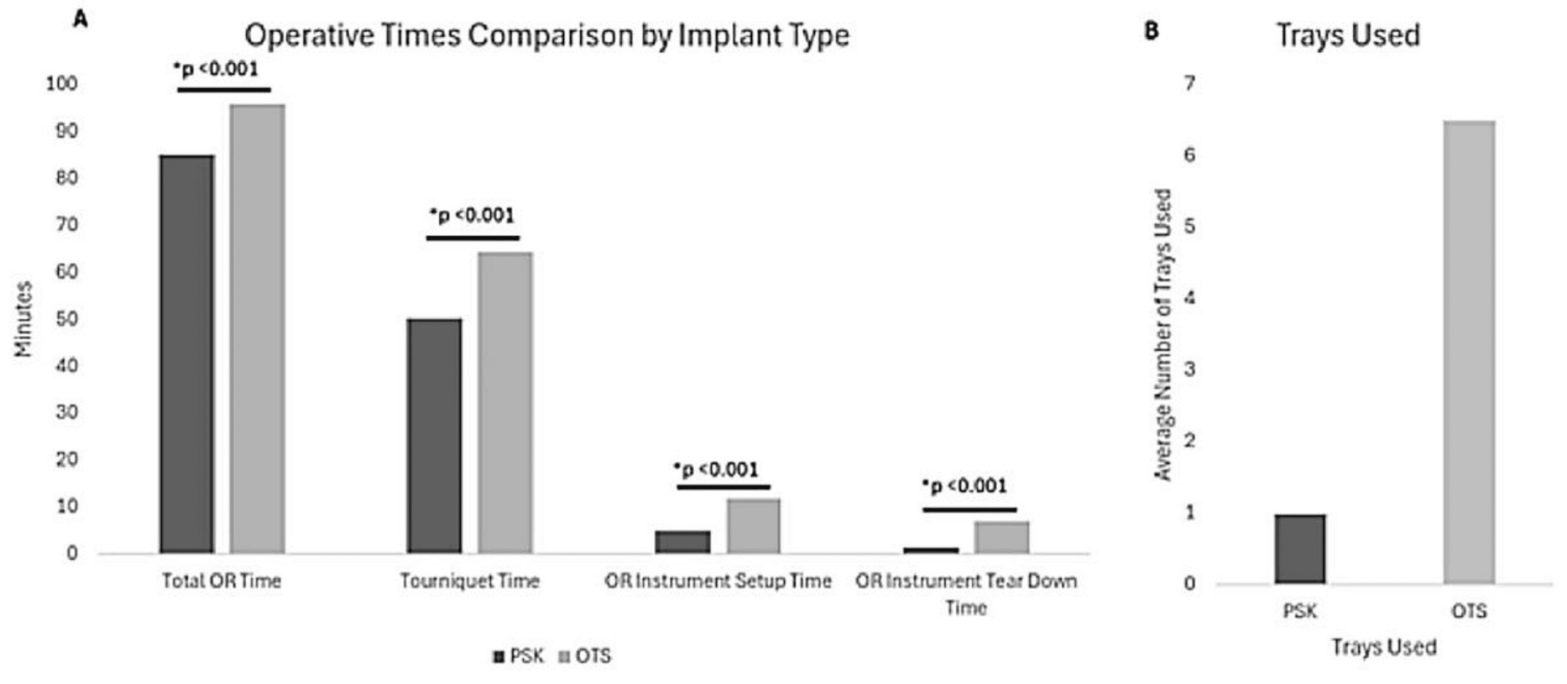
Operative data. A. Comparison of operative times between PSK and OTS systems. B. Comparison of trays used between PSK and OTS groups. * Indicates a statistically significant difference (p < 0.001). Total OR time is defined as wheels in to wheels out and is not the cumulative total of tourniquet time, OR instrument setup time, and OR instrument tear down time. OTS, Off-the-Shelf; PSK, Patient-Specific Knee; OR, Operating room..

Significant differences were observed in operative times. The total operating room (OR) time (wheels in to wheels out) for the PSK group was significantly shorter, averaging 85.2 minutes, compared to 95.9 minutes for the OTS group (p < 0.001). Furthermore, the PSK group showed a notable reduction in tourniquet time (50.2 vs. 64.3 minutes, p < 0.001), OR instrument setup time (5.0 vs. 11.9 minutes, p < 0.001) and OR instrument tear down time (1.5 vs. 7.0 minutes, p < 0.001). The PSK system used less trays than the OTS group (1.0 vs. 6.5 trays, p < 0.001) (Figure 2). These data are summarized in Table 2 and Figure 3. 

**Table 2:** Operative data. This table is based on procedure-level data. ^a^Total OR time is defined as wheels in to wheels out and is not the cumulative total of tourniquet time, OR instrument setup time, and OR instrument tear down time. df, degrees of freedom; OR, Operating room; OTS, Off-the-shelf; SD, Standard Deviation; PSK, Patient-specific knee.

	PSK (N=137)	OTS (N=80)	X^2^(df) = value, P-value	Effect size
Operative laterality			X^2^(1)=0.0002, p=0.988	0.001
Left (%)	67 (48.9)	40 (50.0)	-	-
Right (%)	70 (51.1)	40 (50.0)	-	-
Average operative times in minutes				
Total OR time^a ^(SD)	85.2 (13.4)	95.9 (13.9)	<0.001	
Tourniquet time (SD)	50.2 (15.3)	64.3 (8.02)	<0.001	
OR instrument setup time (SD)	5.0 (7.71)	11.9 (5.61)	<0.001	
OR instrument tear down time (SD)	1.5 (4.97)	7.0 (2.40)	<0.001	
Instrumentation				
Average trays used (SD)	1 (0.00)	6.5 (0.66)	<0.001	

Outcomes data

**Figure 4:**
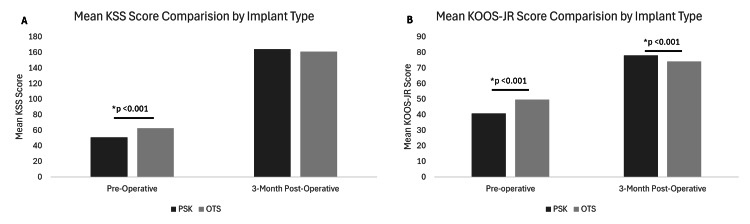
Outcome data. A. KOOS-JR comparison between PSK and OTS groups preoperative and approximately three-months postoperatively. B. KSS comparison between PSK and OTS groups preoperative and approximately three-months postoperatively. * Indicates a significant difference (p < 0.001). KOOS-JR, Knee injury and Osteoarthritis Outcome Score for Joint Replacement; KSS, Knee Society Score; OTS, Off-the-shelf; PSK, Patient-Specific Knee.

**Figure 5:**
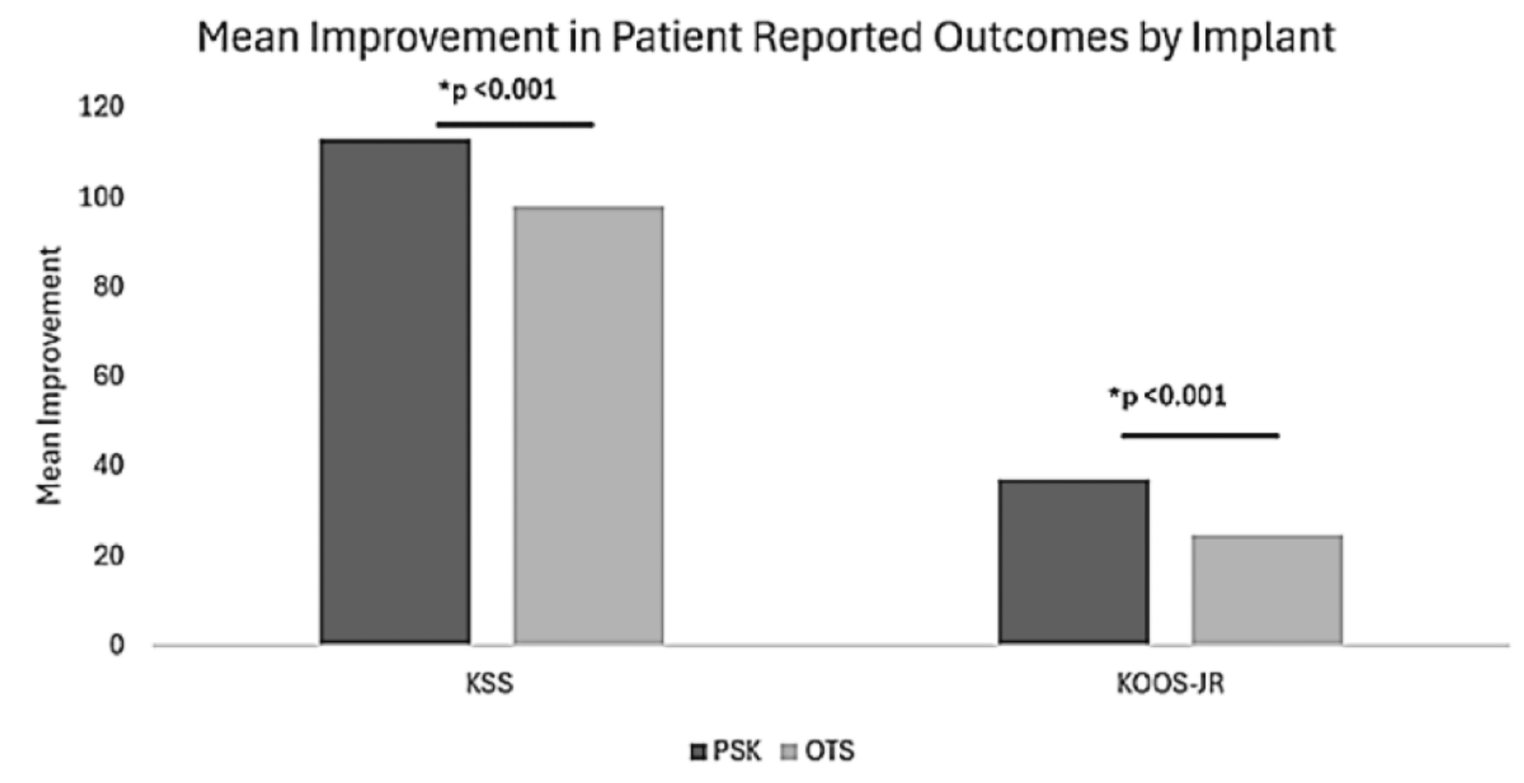
Temporal change in outcome data. Comparison of the difference in outcome data between the PSK and OTS groups over time. * Indicates a significant difference (p < 0.001). OTS, Off-the-shelf; PSK, Patient-specific knee.

The KOOS-JR and KSS were used to assess patient-reported outcomes. At preoperative baseline, participants receiving the PSK had a worse mean KOOS-JR score compared to the OTS group (40.7 vs. 49.6, p <0.001). At the 3-month post-operative visit, the mean KOOS-JR score in the PSK cohort was significantly better than the OTS group (78.1 vs. 74.2, p < 0.001). Preoperatively, mean KSS scores were worse in the PSK group than the OTS group (50.9 vs. 62.7, p < 0.001). At three months, there was no significant difference between mean KSS scores between the groups (164 vs. 161, p = 0.380). There was a significant improvement in both KSS (113 vs. 98.1, p = <0.001) and KOOS-JR (37.3 vs. 24.6) scores over time in the PSK group compared to the OTS group. These data are summarized in Table 3, Figure 4, and Figure 5.

**Table 3:** Average outcomes measurement scores. This table is based on procedure-level data. KOOS-JR, Knee injury and Osteoarthritis Outcome Score for Joint Replacement; KSS, Knee Society Score; OTS, Off-the-shelf; SD, Standard Deviation; PSK, Patient-specific knee.

Preoperative visit	PSK (N=137)	OTS (N=80)	P-value
KOOS-JR total (SD)	40.7 (9.49)	49.6 (11.54)	<0.001
KSS total score (SD)	50.9 (12.00)	62.7 (9.26)	<0.001
3-month post-operative visit	PSK (N=134)	OTS (N=79)	
KOOS-JR total (SD)	78.1 (7.15)	74.2 (10.64)	<0.001
KSS total score (SD)	164.1 (9.52)	160.9 (13.00)	0.038
Difference in measurement scores	PSK (N=134)	OTS (N=79)	P-value
Delta KSS (SD)	113.1 (14.12)	98.1 (14.37)	<0.001
Delta KOOS-JR total (SD)	37.33 (11.16)	24.61 (11.63)	<0.001

Of the 13 (13/217, 6.0%) adverse events, nine (9/137, 6.6%) were in the PSK group and four (4/80, 5.0%) were in the OTS group. Of the nine adverse events in the PSK group, four (4/9, 44%) adverse events were medically managed prior to discharge, and five (5/9, 55%) were serious adverse events resulting in hospital readmission. Of the four adverse events in the OTS group, one (1/4, 25%) resulted in an emergency department visit, and three (3/4, 75%) were serious adverse events resulting in hospital readmission. These data are summarized in Table 4.

**Table 4:** Summary of safety events. ^a^Duration of time that elapsed from the time of the index procedure. N/a represents safety events that occurred at the time of surgery. OTS, Off-the-shelf; ORIF, Open reduction internal fixation; PSK, Patient-specific knee; TKA, Total knee arthroplasty; PCL, Posterior cruciate ligament; SIADH, Syndrome of inappropriate antidiuretic hormone secretion.

Event no.	Cohort	Readmission	Time to event^a^	Description of event
1	PSK	No	n/a	SIADH with elevated sodium levels
2	PSK	No	n/a	Cellulitis
3	PSK	No	n/a	Cellulitis
4	PSK	No	n/a	Deep vein thrombosis
5	PSK	Yes	6 wks	Manipulation of PSK implant while under anesthesia for TKA of the contralateral knee
6	PSK	Yes	8 mo	Revision TKA to address a PCL tear and joint subluxation following a fall
7	PSK	Yes	6 wks	Cardiac catheterization
8	PSK	Yes	10 wks	Cellulitis requiring antibiotics and observation
9	PSK	Yes	4 wks	Renal failure secondary to dehydration
10	OTS	No	1 wk	Cellulitis
11	OTS	Yes	2 wks	Quadricep tear repair
12	OTS	Yes	1 wk	Periprosthetic fracture of the distal femur requiring ORIF followed by distal femoral replacement
13	OTS	Yes	1 wk	Removal and replacement of the TKA implant with an antibiotic spacer

## Discussion

Patient-specific benefits

The increased use of three-dimensional imaging and advanced manufacturing has accelerated the development of patient-specific instrumentation (PSI) and patient-specific implants for arthroplasty. Ultimately, these advances have improved operating room efficiency, postoperative alignment, and long-term outcomes [8]. In the present study, the improved functional outcomes and patient satisfaction scores (KOOS-JR and KSS) at the three-month follow-up visit for the PSK group compared to the OTS group may be attributed to the precise alignment and kinematics offered by patient-specific implants [9,10]. León-Muñoz et al. discuss the potential of PSI in TKA to improve surgical effectiveness and efficiency [11]. They note that while PSI may not significantly increase accuracy over conventional methods, it offers advantages such as reduced operative time and perioperative blood loss, while maintaining similar clinical outcomes. Other groups have reported lower rates of surgical site infections, wound dehiscence, and transfusion requirements with reduced operative times in TKA [12].

Furthermore, Camarda et al. provide a comprehensive review of the literature on PSI, highlighting its role in reducing the complexity of conventional alignment and sizing tools, which can contribute to improved operative room efficiency and post-operative alignment [8]. In the present study, we found that the use of PSI led to significantly decreased total operating time, tourniquet time, and incision-to-implant time. Ravi et al. described a median surgical duration of 106 minutes among 92,343 primary TKA patients, and Tompkins et al. reported a mean OR time of 139 minutes in robotic-assisted TKA across approximately 2400 surgeries [13,14]. While mean total OR time in both surgical groups in our study (85.2 vs. 95.9 minutes) was faster than the mean total OR time reported by Ravi et al. and Tompkins et al., the PSK system was approximately 10% faster than the OTS system in our study [13,14]. Moreover, in comparison to robotic-assisted TKA, PSK TKA procedures do not require the additional set up time, personnel requirements, and capital expenditures that are required for a surgical robotic system [15]. These findings underscore the need for a balance between technology-assisted precision and surgeon expertise in patient-specific TKAs. The use of preoperative planning and intraoperative flexibility could explain the observed patient benefits in our study, such as reduced operative times without compromising the quality of outcomes.

Economic implications

In addition to the positive health outcomes that are associated with reduced OR time, economic benefits exist as well. Healthcare spending in the United States is the highest in the world and is projected to increase to 6.2 trillion dollars by 2028 [16]. As the surgical volume and associated healthcare spending continue to grow for TKA, payers have continued to seek viable strategies to reduce costs [17,18]. The increased operative efficiency seen with the PSK system compared to the OTS system may improve resource utilization, increase patient throughput, and enhance surgeon productivity. This improvement in surgical duration aligns with the findings of Healy et al., who reported that the implementation of clinical pathways and knee-implant standardization programs at a hospital led to a 19% reduction in hospital costs, along with a decrease in the average length of hospital stay from 6.79 days to 4.16 days, demonstrating that systematic approaches can significantly reduce costs without compromising patient outcomes [19]. In a review by Smith et al., the mean cost of operating room time was $46.04 ± $32.31 per minute [20]. Based on the data presented in this study, facilities could save approximately $146.91 - $838.35 ($492.63 ± $345.72) per case with the PSK vs. OTS systems when comparing total OR time. Additionally, research by Garbarino et al. indicates that longer operative times in revision TKA are significantly associated with longer hospital stays, suggesting that efficiencies in operative time can lead to economic savings [21]. These studies collectively emphasize the importance of optimizing surgical efficiency in TKA to manage healthcare costs effectively while maintaining high-quality patient care.

Optimizing the number of surgical trays to improve operating room efficiency and reduce instrument processing fees is another viable strategy for cost containment. In a study by Lonner et al., 1277 TKAs were analyzed by recording instrument types, instrument numbers, and sterilization process time [22]. After optimizing the trays by removing redundant or underutilized instruments, only 59 (67.8%) of the original 87 instruments remained for TKA, allowing all necessary instruments to be stored in one tray. This optimization reduced the mean set-up time from 20.7 to 14.2 minutes, resulting in a time saving of 40-75 minutes in the sterilization process, and an average annual savings of $191,434.88. Sterilization costs vary from institution but broadly fall within the $0.51 to 11.52 range per instrument [22-24]. In the present study, the PSK system uses one tray compared to an average 6.5 trays used in the OTS system. These results imply that significant cost reduction can be obtained when using the PSK system by reducing total OR time and the number of instrumentation trays.

Based on estimation of OR time costs of $46.04/minute, tray management logistics of $85/tray, and tray re-sterilization costs of $70/tray, the present study estimates an average savings of $492.20 in OR time costs, $467.50 in tray management, and $385 in tray sterilization costs when using the PSK system as compared to the OTS TKA [20,25,26]. Click or tap here to enter text. This combined savings of $1,344.70 per TKA performed is meaningful to the overall economic burden of TKAs, particularly at high volume centers. In large facilities, thousands of TKAs are performed annually. Based on these calculations, facilities would save $1,344,700.00 per 1000 TKAs performed.

Readers may argue that the use of a PSK system requires additional time and costs associated with preoperative planning that otherwise would not be required for use of an OTS implant. It is important to note that the PSK system in this study did not add any additional burden. To initiate the PSK design process, surgeons submit a preoperative CT scan and an implant order. The ordering process takes approximately five to 10 minutes, based on system familiarity. Unlike with other patient-specific products, the surgeon does not need to review the design plan, and instead, the manufacturer completes all required subsequent steps. First, the CT scan is converted to a 3D model which is used to create the implant design. Next, the implant is manufactured. Patient-specific instrumentation is then created to match the PSK implant. After the implant and instruments pass inspection, components are shipped together, in a single tray, arriving at the facility approximately one week prior to the surgery date. While more planning is required to produce a patient-specific implant, this process is seamless and managed by the manufacturer. Preoperative planning also negates the need for utilizing sizing guides intraoperatively, which is commonly used when implanting OTS devices, ultimately saving OR time. When compared to robotic-assisted surgery, a preoperative CT scan is similarly required for use of the Mako robotic system (Stryker; Kalamazoo, MI), the industry gold standard. The Mako robotic system costs roughly $1,000,000 with an additional $160,000 in annual maintenance fees, resulting in exorbitant costs [27]. In addition to costs associated with the initial purchase and routine maintenance, Christen et al. reported that robot-assisted surgery resulted in a 14-minute increase in surgery time, driving costs even higher [28]. While imageless robotic-assisted surgery reduces preoperative CT scan costs, these saved costs are nullified by surgery time nearly doubling to 25 minutes [28].

Limitations and future directions

While the results of our study are promising, several limitations must be acknowledged. The observational design of the study limits the ability to establish causality. While the focus of this study was to report on the economics of patient-specific versus OTS systems, the relatively short follow-up period restricts our understanding of the long-term effects and durability of the PSK system in comparison to the OTS system. Prior long-term studies of the PSK system have comprehensively assessed the durability and long-term cost-effectiveness of these systems demonstrating favorable clinical outcomes and patient satisfaction [29,30]. Furthermore, the study's patient population may not fully represent the diversity seen in broader clinical practice. Future research should focus on inclusion of more diverse populations to establish the generalizability of the findings. The total number of knees implanted and patient demographics within each group (OTS, PSK) were also not perfectly matched. The PSK cohort had younger patients who preoperatively scored worse on patient reported outcomes. The younger average age of the PSK cohort coupled with poorer baseline patient reported outcomes may have resulted in larger improvements in postoperative outcome scores, and may not be a reflection of the type of implant utilized. Additionally, the integration of PSI in TKA, while promising, warrants further investigation. Studies such as those conducted by León-Muñoz et al. have explored the advantages and limitations of PSI, highlighting the need for continuous evaluation of this technology [11]. While PSI may offer benefits in operative time and efficiency, its impact on long-term patient outcomes and cost savings is still to be fully understood. Future directions in this field should include long-term randomized controlled trials, studies with diverse patient populations, and investigations into the evolving role of technological advancements in TKA.

## Conclusions

The PSK system was associated with considerable benefits in terms of operative efficiency, patient-reported outcomes, and potential cost savings relative to a standard OTS system. Although future studies are needed to establish long-term benefit, these findings contribute to the growing body of evidence supporting the adoption of patient-specific implants and instrumentation in TKA. More importantly, PSK implants and instrumentation allow for the surgical precision that is achieved with robot-assisted TKA but has the added benefits of minimizing financial burden through reduced OR time and no required initial machine purchase or annual maintenance fees. Other benefits include reducing postoperative complications by reducing overall surgery time (less blood loss, lower risk of infection, lower rate of compromised trays).
